# Observation of Boron Vacancy Concentration in Hexagonal Boron Nitride at Nanometer Scale

**DOI:** 10.1021/acs.nanolett.5c02988

**Published:** 2025-08-20

**Authors:** Jun Kikkawa, Chikara Shinei, Jun Chen, Yuta Masuyama, Yuichi Yamazaki, Teruyasu Mizoguchi, Koji Kimoto, Takashi Taniguchi, Tokuyuki Teraji

**Affiliations:** † 52747 National Institute for Materials Science, 1-1 Namiki, Tsukuba 305-0044, Japan; ‡ National Institutes for Quantum Science and Technology, 1233 Watanukimachi, Takasaki 370-1292, Japan; § Institute of Industrial Science, 13143 The University of Tokyo, 4-6-1 Komaba, Meguro, Tokyo 153-8505, Japan

**Keywords:** hexagonal boron nitride, boron vacancy, electron energy loss spectroscopy, scanning transmission electron microscopy, frist-principles simulation

## Abstract

Negatively charged boron vacancy (V_B_^–^) ensembles in hexagonal boron nitride (h-BN) have attracted considerable attention as a promising platform for quantum sensing. Current challenges include the experimental validation of the spatial distribution and electronic states of optically active V_B_^–^ and optically inactive neutral boron vacancy (V_B_^0^) defects. To address these issues, we employ electron energy loss spectroscopy (EELS) combined with scanning transmission electron microscopy (STEM) using monochromated 30-keV electrons, effectively reducing background interference. This approach unveils distinct spectral peaks at 2.5 and 1.9 eV, corresponding to V_B_^–^ and V_B_^0^ defects, respectively. Furthermore, we achieve nanometer-scale concentration mapping for V_B_^–^ and V_B_^0^ defects, advancing insights into spin defect configurations crucial for optimizing quantum sensor performance.

## Graphical abstract

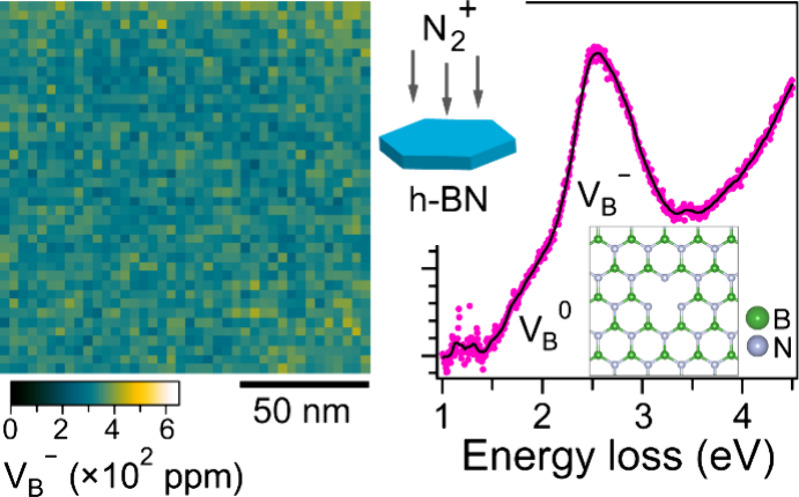

Quantum sensors hold considerable potential for measuring a wide range of physical and chemical properties, providing exceptional resolution and precision.^1,2^ Ensembles of color centers (i.e., point defects caused by vacancies and impurities) in hexagonal boron nitride (h-BN) are promising platforms for next-generation quantum sensors as post negatively charged nitrogen-vacancy (NV^–^) ensembles in diamond.^3,4^ h-BN is a two-dimensional material with a wide bandgap at ∼ 6 eV,^5,6^ and has the advantages of easy integration into quantum devices, high photon extraction efficiency, and photon wavelength diversity.^7,8^ Negatively charged boron vacancies (V_B_^–^), antisite nitrogen vacancies (N_B_V_N_), and carbon-based defects are color centers generating luminescence at ∼1.5,^9–13^ ∼2.0,^14,15^ and ∼4.1 eV,^16–20^ respectively. In particular, the V_B_^–^ centers exhibit spin-dependent photon emission at room temperature, a desirable property for quantum sensing.^9–13^ The spatial V_B_^–^ distribution uniformity, V_B_^–^ concentration, and the distance between individual V_B_^–^ defects are fundamental properties, in addition to the crystallinity and purity of h-BN, for determining spatial resolution and sensitivity in quantum sensors. Ion or electron irradiation on h-BN generates V_B_^–^ defects,^9–13^ and can simultaneously generate neutral born vacancies (V_B_^0^) and other types of defects,^21–27^ which coexist with intrinsic impurity-related defects.^14–20,28^ Direct observations have shown that electron and He^+^ ion irradiation preferentially generate V_B_ defects rather than V_N_ defects.^25–27^ However, the distribution, concentration, and spacing of V_B_^–^ and V_B_^0^ defects are as yet not fully understood because of the absence of a standardized measurement method at the nanometer scale, especially for optically inactive V_B_^0^. Furthermore, despite the first-principles simulations of the electronic structures of V_B_^–^ and V_B_^0^ defects,^17,29–31^ experimental validation remains necessary. Therefore, it is crucial to measure the arrangement and electronic states of V_B_^–^ and V_B_^0^ defects.

Electron energy loss spectroscopy (EELS) combined with scanning transmission electron microscopy (STEM) enables probing the subgap states of lattice defects and the chemical bonding states at defect sites.^32–35^ However, measuring the detailed EELS spectral structure from point defects in bulk crystals presents a significant challenge, because of the low concentration of point defects (≲ 10^3^ ppm) and the weakness of EELS signals compared with other intrinsic signals. Three types of background intensity can hinder the detection of EELS signals of defect states. The first is the tail of the zero-loss peak (ZLP, i.e., elastic scattering peak), whose signal can become dominant even in several eV region in EELS. A smaller energy spread of the incident electrons evaluated with full width at half-maximum (fwhm) or more appropriately at 10^–*n*^ (*n* ≥ 1) of the ZLP,^36^ is required to reduce the contribution of the ZLP tail. The second is EELS signals, which can appear at around several eVs owing to the generation of Cherenkov radiation.^37^ This radiation can be reduced or ignored by lowering the energy of incident electrons or by using a thinner specimen.^32,33,38^ The third is detector noise. It is very important to reduce readout noise in addition to gain noise so that weak signals are not buried in their noise.

In this study, to measure electronic states of V_B_^–^ and V_B_^0^ defects in h-BN by EELS, we use monochromated 30-keV electrons, reducing the fwhm of the ZLP to 40 meV,^39^ and suppressing Cherenkov radiation.^32,33,38,40^ To detect scattered electrons in EELS, we use a charge-coupled-device (CCD) camera with a high-sensitivity scintillator optimized for 30-keV electrons,^39^ and the readout noise reduction scheme.^41,42^ EELS in combination with first-principles simulations revealed a high peak at 2.5 eV with enhanced intensity appearing at the shoulder position of 1.9 eV, which are respectively assigned to signals from V_B_^–^ and V_B_^0^ defects. Furthermore, we obtain the concentration maps for V_B_^–^ and V_B_^0^ defects at the nanometer scale.

h-BN single crystals were grown using a temperature gradient method at a high pressure,^43^ and their flakes with thicknesses of 30–200 nm were prepared using a tape-peeling method (details of the specimen preparation and experimental methods are described in Supporting Information). The h-BN flakes were dispersed on a holey carbon-film-supported copper grid and then irradiated with a 40-keV nitrogen ion (N_2_^+^) beam along the *c*-axis at a total dose of 1 × 10^15^ cm^–2^ at room temperature,^11^ as shown in Figure 1a. Figure 1b shows the photoluminescence (PL) spectra of the pristine and irradiated h-BN flakes at room temperature with an incident photon energy of 2.33 eV. PL occurs with a peak maximum at 1.53 eV (=810 nm) only after the irradiation, indicating the formation of optically active V_B_^–^ defects, as observed in previous studies.^9–13^ Figure 1c shows the optically detected magnetic resonance (ODMR) spectrum of the irradiated h-BN flake. ODMR occurs at a microwave frequency of ∼3.5 GHz after the irradiation, corresponding to a ground state zero-field splitting between spin states *m*_s_ = 0 and *m*_s_ = ±1 for V_B_^–^ in h-BN.^4,9^ Figure 1d displays the EELS spectra of the pristine and irradiated h-BN flakes below 6.2 eV. Two characteristic EELS intensities appear in the subgap region only for the irradiated h-BN flake as indicated by the arrows (Figure 1d). One is an asymmetric signal centered at ∼2.5 eV and the other is a continuous intensity distribution ranging from 3.8 to 5.9 eV. These EELS intensities reflect the density of states (DOS) of the defect levels introduced by the irradiation. The asymmetric 2.5 eV signal was more clearly observed in flakes thicker than ∼100 nm. In the range of 6–30 eV, there is no marked difference in EELS profiles between the pristine and irradiated h-BN flakes (Figure S1a). In addition, no marked changes were observed at the N *K* edge after the irradiation; only a slightly asymmetric broadening of the peak at 191.8 eV was detected at the B *K* edge (Figures S1b and S1c), suggesting the formation of nitrogen vacancies (see details in Supporting Information).^44^ We also observed cathodoluminescence (CL) at approximately 4.1 eV (Figure S2), which occurred depending on the measurement position for both pristine and irradiated flakes. This indicates the presence of carbon-related defects in the original h-BN crystal.^16–20^ As the energy levels associated with these defects lie outside the energy range of interest (i.e., 1.5–3.5 eV), it is appropriate to focus exclusively on the V_B_^–^ and V_B_^0^ defects hereafter (see details in Supporting Information).

**Figure 1:**
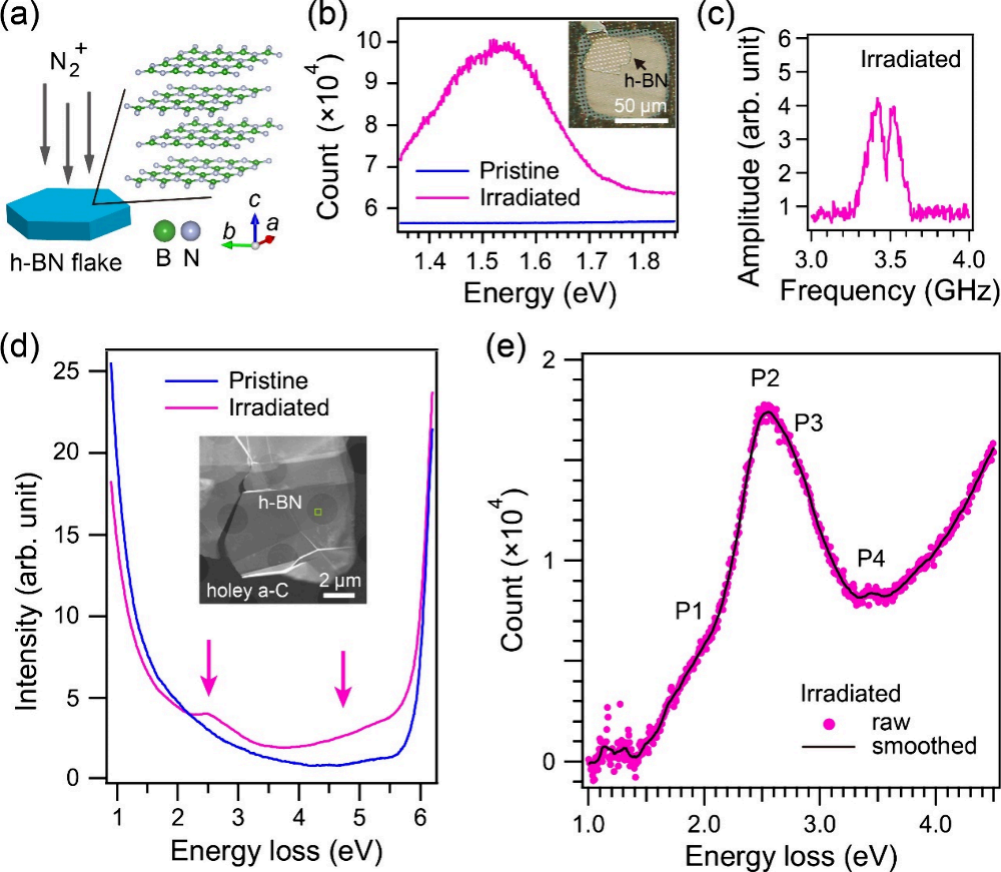
(a) Illustration of N_2_^+^ ion irradiation of h-BN flake. (b) PL spectra of pristine and irradiated h-BN flakes. (c) ODMR spectrum of irradiated h-BN flake. (d) EELS spectra of pristine and irradiated h-BN flakes. The inset optical microscopy and annular dark-field STEM images in (b) and (d) respectively show the h-BN flakes on a holey a-C film. (e) EELS spectrum after subtraction of the ZLP tail for the irradiated h-BN flake in (b), revealing four characteristic intensity peaks labeled P1–P4. Row data (solid circle) and smoothed profile (solid line).

To identify fine structures of the 2.5 eV asymmetric signal in Figure 1d, we conducted EELS with a high energy resolution (i.e., fwhm of the ZLP, 40 meV). By scanning the electron probe in steps of 3.6 nm in 143 nm-square areas in the irradiated h-BN flake, we obtained 1600 single EELS spectra. By summing the single spectra and subtracting the background intensity mainly due to the ZLP tail with a power-low fit (Figure S3), we found that the asymmetric 2.5 eV signal is composed of four characteristic intensities, as shown in Figure 1e. The high intensity peak P2 locates at 2.5 eV, overlapping with relatively low intensity peaks labeled P1 at 1.9 eV, P3 at 2.9 eV, and P4 at 3.45 eV. The appearance of the intense signal at 2.5 eV in EELS is consistent with the maximum absorption at ∼2.6 eV in PL excitation measurements for V_B_^–^ defects in h-BN.^10^ Note that the asymmetric 2.5 eV signal is weaker than intrinsic bulk signals. For instance, the EELS intensity of the 2.5 eV signal (i.e., integrated intensity in the range of 1.34–3.34 eV) is approximately 10^–1^ of that of optical phonons (i.e., integrated intensity in the range of 0.13–0.28 eV), despite the 13-fold difference in integration range (Figure S4).

To understand the fine structures of the 2.5 eV asymmetric signal in Figure 1e, we conducted first-principles simulations (details of the calculation methods are described in Supporting Information). Figures 2a–2c show the electronic DOS values of h-BN crystals without defects, with a V_B_^–^ defect, and with a V_B_^0^ defect, respectively (see Figure S5 for a wider energy range). Occupied and unoccupied states are filled and blank, while up- and down-spin states are displayed in the upper and lower side, respectively. The three DOS diagrams are aligned with the 2s levels (Figure S5). The positions of VBM and CBM in Figures 2b and 2c denote those for the perfect crystal (Figure 2a). As shown in Figures 2b and 2c, subgap states between VBM and CBM differ largely depending on whether the charge of V_B_ is – 1 or zero. The diagonal components of the dielectric tensor perpendicular and parallel to the *c*-axis are written as **ε**_*xx*_ = ε_1,*xx*_ + *i*ε_2,*xx*_(=**ε**_*yy*_) and **ε**_*zz*_ = *ε*_1,*zz*_ + *i*ε_2,*zz*_, respectively. Figures 2e–2g show the imaginary parts ε_2,*xx*_ and ε_2,*zz*_ for the perfect crystal, the V_B_^–^ defect, and the V_B_^0^ defect, respectively (see Figure S6 for a wider energy range and real parts). The imaginary parts represent absorption. Note that *ε*_2,*xx*_ for the V_B_^–^ defect is intense with fine structures denoted as A–F in the 2.0–4.0 eV range (Figure 2f). This indicates that electronic excitations at 2.0–4.0 eV in the *ab*-plane direction are dominant compared with those along the *c* **-**axis. For the V_B_^0^ defect, *ε*_2,*xx*_ has a relatively low intensity and fine structures denoted as G–J in the 0.5–2.5 eV range (Figure 2g), whereas there is no characteristic intensity in *ε*_2,*xx*_ and *ε*_2,*zz*_ for the perfect crystal because of the absence of subgap states (Figure 2e). The A–F peaks in Figure 2f and the G–J peaks in Figure 2g originate from the electron excitations between subgap states, A–F in Figure 2b and G–J in Figure 2c. The loss function *L* when the electron incident direction is parallel to the *c*-axis and the convergence angle α = 0 is calculated as^45^

$$L=\mathrm{-I}m\left[\frac{1}{2\varepsilon_{zz}}\;\ln\left(1+\frac{\varepsilon_{zz}\beta^{2}}{\varepsilon_{xx}{\theta_{E}}^{2}}\right)\right]$$

θ_E_ = Δ*E*/2*E*_0_ in nonrelativistic form, where *E*_0_ (=30 keV) and Δ*E* respectively represent the incident energy and energy loss of the primary electron: θ_E_ = 4.2 × 10^–2^ mrad for Δ*E* = 2.5 eV. Considering that the electron probe used in STEM–EELS has α (≠0), β is approximately replaced with β* = $\sqrt{\alpha^{2}+\beta^{2}}$ to calculate *L* (details are described in Supporting Information). Figures 2h–2j show *L* for the perfect crystal, the V_B_^–^ defect, and the V_B_^0^ defect, respectively. The profile of *L* mainly reflects that of ε_2,*xx*_. The A′–F′ peaks in Figure 2i and the G′–J′ peaks in Figure 2j reflect the A–F peaks in Figure 2f and the G–J peaks in Figure 2g, respectively. This is because ε_2,*xx*_ in *L* becomes dominant when θ_E_ ≪ β and also because ε_2,*zz*_ is originally small. Reflecting the intensities of ε_2,*xx*_ in *L* for the V_B_^–^ and V_B_^0^ defects (Figures 2f and 2g), the intensity of *L* for the V_B_^–^ defect [*L* (V_B_^–^)] in the range including the A′–F′ peaks (Figure 2i) is higher than that *L* for the V_B_^0^ defect [*L* (V_B_^0^)] in the range including the G′–J′ peaks (Figure 2j). Figures 2i and 2j also suggest that the EELS intensity in the 4–6 eV range in Figure 1d originates from other types of defects and partly from the V_B_^–^ defects. Figure 3 shows plots of *L* (V_B_^–^) and *L* (V_B_^0^) with coefficients of 0.16 and 0.84, respectively, and their linear combination, 0.16 *L* (V_B_^–^) + 0.84 *L* (V_B_^0^), where *L* was plotted so that the linear combination profile matched the smoothed EELS spectrum (Figure 1e) (see Figure S8 for the method of determining the coefficients). The good agreement between the linear combination and the EELS spectrum enables the assignment of the origin of the P1–P4 peaks in EELS and reveals the concentration ratio of the V_B_^0^ defects to the V_B_^–^defects, V_B_^0^/V_B_^–^ is 5.3 as the average value within the measured 143 nm-square area. The intense P2 peak in EELS corresponds to the A′ and B′ peaks in *L* (V_B_^–^), and, thus, the electron excitations A and B in Figure 2b. The P3 and P4 peaks correspond to the C′ and E′ peaks in *L* (V_B_^–^), whereas the P1 peaks correspond to the I’ peak in *L* (V_B_^0^). The finding that the optically inactive V_B_^0^ coexists with the optically active V_B_^–^ implies that adjusting the charge state from 0 to – 1 can increase the V_B_^–^ concentration through electron injection.^22,46^ Figure 4 shows a schematic unifying our understanding from the results of PL, EELS, and first-principles simulations in this study and PL excitation in a previous study for V_B_^–^.^10^ As illustrated in the DOS schematics of the ground state for V_B_^–^ (Figure 4), the 1.53 eV PL occurs as an electronic relaxation process after the 2.5 eV excitation (i.e., absorption in EELS and PL excitation) between the occupied and unoccupied defect states. The remaining energy of approximately 1 eV is attributed to nonradiative relaxation.

**Figure 2:**
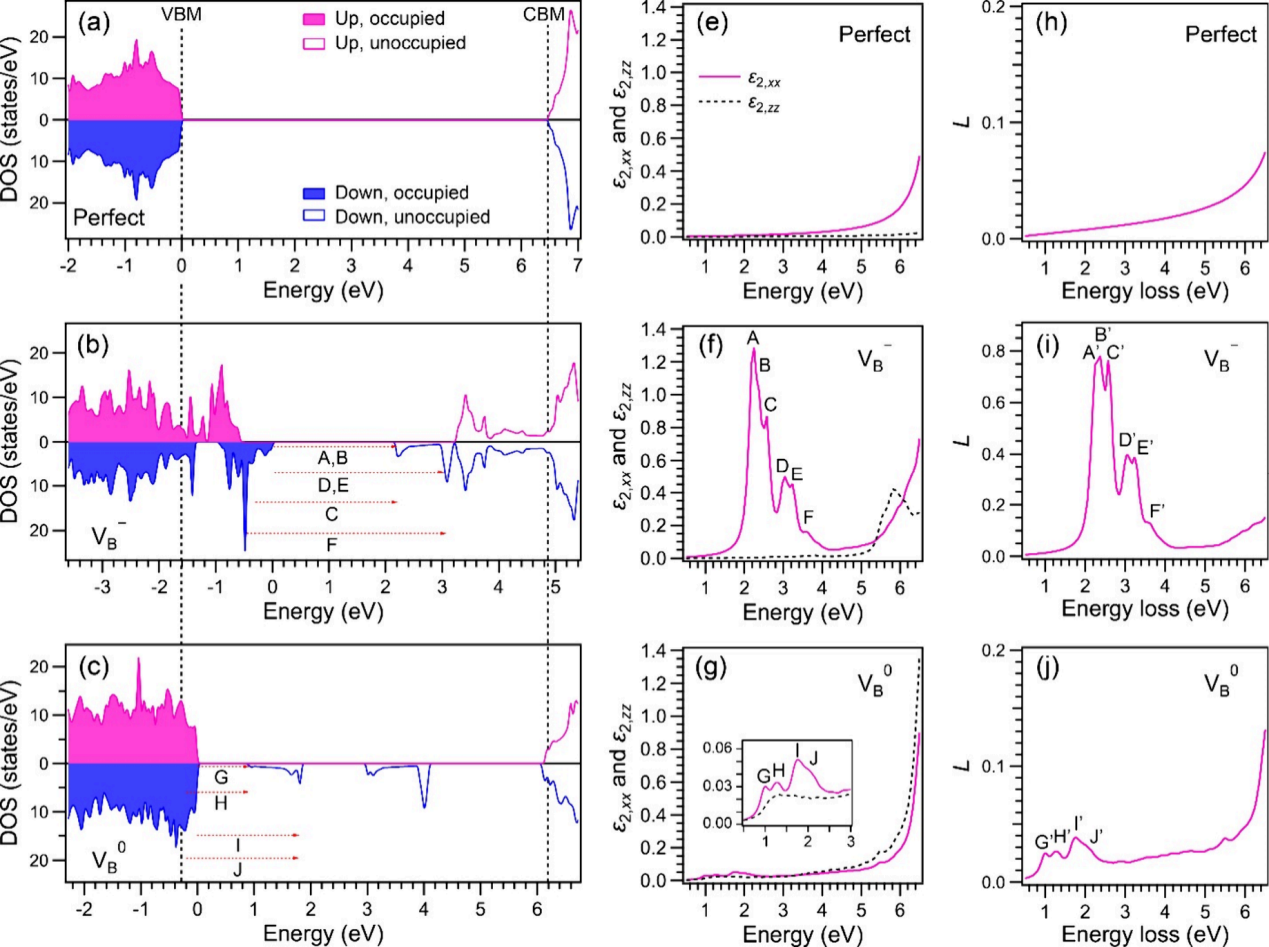
First-principles simulations. [(a)–(c)] DOS values of h-BN crystals without defects, with a V_B_^–^ defect, and with a V_B_^0^ defect, respectively. The filled and blank areas are the occupied and unoccupied states, respectively. The upper and lower sides are the up- and down-spin states, respectively. [(e)–(g)] Imaginary parts of dielectric function, ε_2,*xx*_ (solid line) and ε_2,*zz*_ (dashed line) for perfect crystal in (e), V_B_^–^ in (f), and V_B_^0^ in (g). Intensities in *ε*_2,*xx*_ denoted by A–F in (f) and G–J in (g) originate from electron excitations indicated by A–F in (b) and G–J in (c), respectively. [(h)–(j)] Loss functions (*L*) for perfect crystal in (h), V_B_^–^ in (i), and V_B_^0^ in (j). Intensities in *L* denoted by A′–F′ in (i) and G′–J′ in (j) predominantly reflect the intensities in ε_2,*xx*_ denoted by A–F in (f) and G–J in (g).

**Figure 3:**
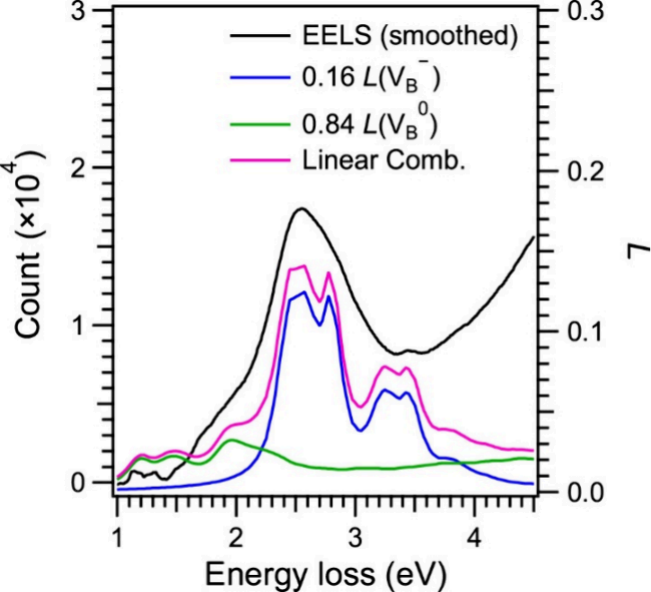
Plots of the loss function *L* for V_B_^–^ (blue) and V_B_^0^ (green) in Figure 2 with factors of 0.16 and 0.84, respectively, their linear combination (magenta), and smoothed EELS spectrum (black) in Figure 1e.

**Figure 4:**
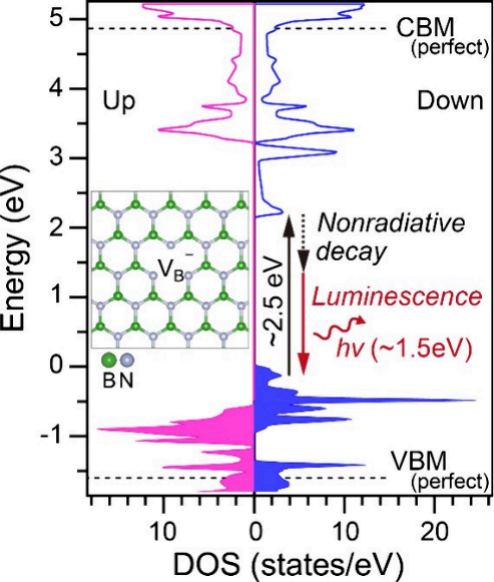
Schematic of the 2.5 eV electron excitation (i.e., absorption) and 1.5 eV luminescence accompanied by nonradiative decay at the V_B_^–^ defect in h-BN, illustrated in the DOS schematic of the ground states.

To evaluate the absolute concentrations of V_B_^–^ and V_B_^0^ by EELS, we utilize the vacancy concentration in the supercell for first-principles simulations, which is 13889 ppm for both V_B_^–^ and V_B_^0^. Using the integrated intensities for V_B_^–^ and V_B_^0^ (i.e., V_B_^–^ + V_B_^0^) in the range of 1.0–3.0 eV and the π-plasmon in the loss functions and those in the EELS spectrum, we estimated the average concentration of V_B_^–^ + V_B_^0^ as approximately 2000 ppm (details are given in Figure S9). We assumed that the intensity ratio of V_B_ to the π plasmon in EELS is proportional to the V_B_ concentration. Then, the average V_B_^–^ and V_B_^0^ concentrations are approximately estimated as 300 and 1700 ppm, using the coefficients 0.16 and 0.84 (Figure 3), respectively. The average V_B_^–^ concentration can be evaluated directly using the integrated intensities for V_B_^–^ in the range of 2.3–3.0 eV, resulting in 300 ppm as well (details are given in Figure S10). By implementing this method for the original 1600 single spectra from the 143 nm-square area (i.e., 40 × 40 pixels), we obtained the concentration map of V_B_^–^ and its histogram, as shown in Figures 5a and 5b, respectively. The V_B_^–^ concentration is nearly uniform without significant segregation (Figure 5a). The Gaussian fit in Figure 5b provides the center and standard deviation of 330 ± 33 ppm, which is close to the average value of 300 ppm estimated above. Figures 5c and 5d respectively show the map of the concentration ratio of V_B_^0^ to V_B_^–^ (i.e., V_B_^0^/V_B_^–^) and its histogram, where the V_B_^0^/V_B_^–^ ratio at each pixel was obtained using the integrated intensities in the ranges of 1.7–2.0 eV for V_B_^0^ and 2.3–2.6 eV for V_B_^–^ (Figure S8a). The V_B_^0^/V_B_^–^ map represents the distribution of the charge state ratio (i.e., 0 to – 1). The negative values of the V_B_^0^/V_B_^–^ ratio for several pixels are due to the excess removal of ZLP tail signals and the low signal-to-nose ratio. The Gaussian fit in Figure 5d gives V_B_^0^/V_B_^–^=5.0 ± 1.7, which closely matches the average V_B_^0^/V_B_^–^=5.3 obtained after integrating the 1600 single spectra described above. By multiplying the V_B_^–^ map (Figure 5a) and the V_B_^0^/V_B_^–^ map (Figure 5c), we also obtained the concentration map of V_B_^0^ and its histogram as shown in Figures 5e and 5f, respectively. The Gaussian fit in Figure 5f provides the center and standard deviation of 1647 ± 648 ppm, which is close to the average value of 1700 ppm estimated above. Figures 5g–5i show plots of typical single EELS spectra at 1 pixel (3.6 nm-square areas) in Figure 5c with the V_B_^0^/V_B_^–^ values of 1.4, 5.0, and 7.1, respectively. The smoothed line profiles certainly reveal the increase in P1 intensity at ∼ 1.9 eV from Figures 5g to 5i. Regarding the spatial resolution of the maps (Figure 5), the diameter and scan step of the electron probe we used were 0.6–0.7 and 3.6 nm, respectively, whereas the effective diameter considering the delocalization of EELS around 2.5 eV was estimated to be 8 nm.^37^ Thus, the maps in Figure 5 are blurred by approximately 2 × 2 pixels compared with the actual intensity distribution.

**Figure 5:**
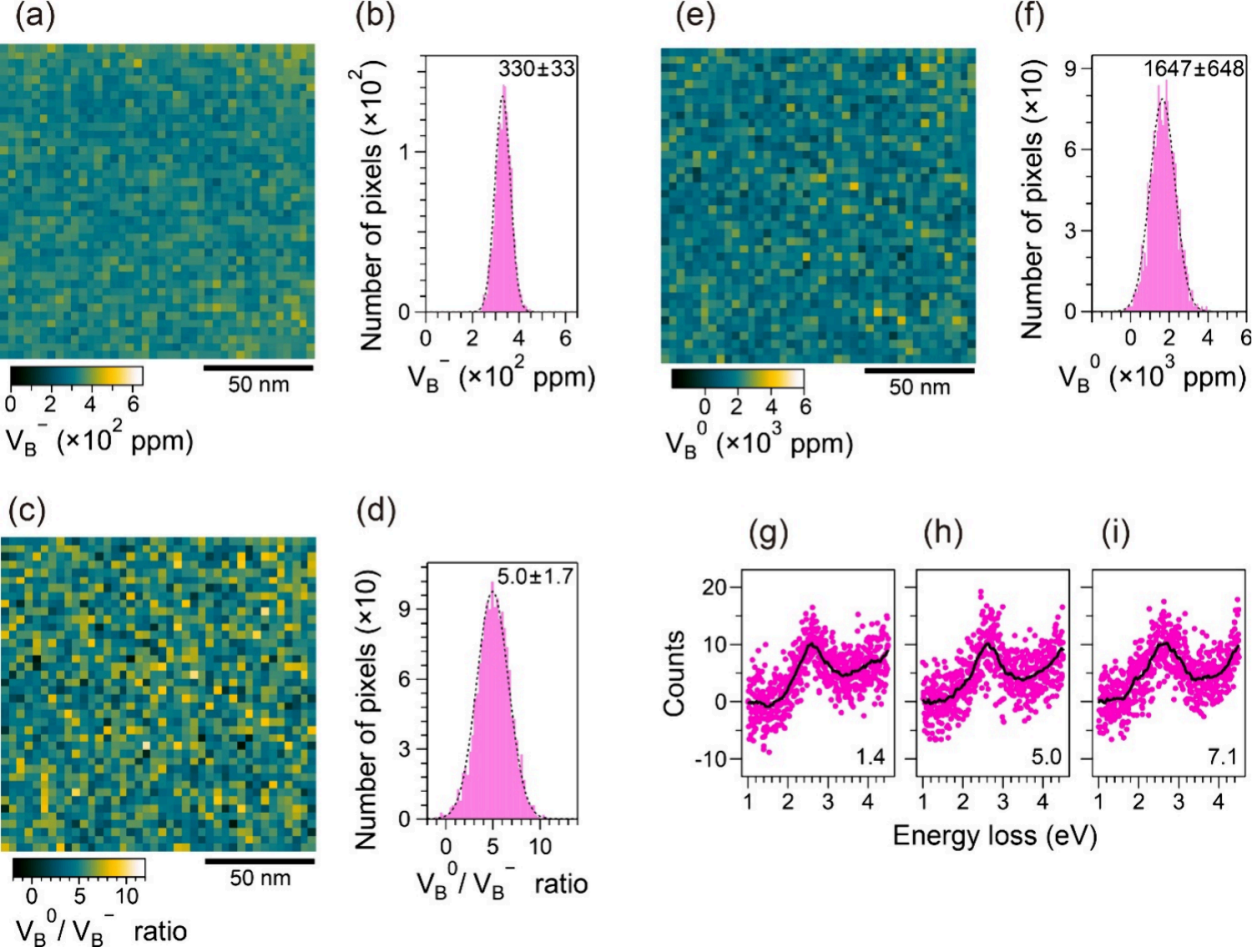
Concentration maps of vacancies in the irradiated h-BN flake. The V_B_^–^ map in (a) and its histogram in (b). The V_B_^0^/V_B_^–^ ratio map in (c) and its histogram in (d). The V_B_^0^ map in (e) and its histogram in (f). All maps have the same area. The centers and standard deviations for Gaussian fits in the histogram are 300 ± 33 ppm, 5.0 ± 1.7, and 1647 ± 648 ppm in (b), (d), and (f), respectively. [g–h] Typical single EELS spectra at 1 pixel (3.6 nm-square areas) with the V_B_^0^/V_B_^–^ ratios of 1.4, 5.0, and 7.1 in (g), (h), and (i), respectively.

Finally, we briefly discuss the quantitative aspects of the concentration maps in Figure 5. The primary concern lies in the comparison between the electron arrival time interval Δ*t* at the electron probe position in STEM–EELS and the lifetime τ from the excited state back to the ground state for V_B_^–^ and V_B_^0^. In this study, Δ*t* was 1.3 ns (i.e., the probe current of ∼120 pA) and the exposure time at each pixel was 0.6 s in Figure 5. The excited state for V_B_^–^ returns to the ground state predominantly via a singlet metastable state.^31,47^ The lifetime of this metastable state is approximately 10–30 ns at room temperature, making τ longer than this,^47–50^ whereas the lifetime of V_B_^0^ remains unknown. This suggests the potential for underestimating the V_B_^–^ concentration during EELS measurement at each pixel. The measurement was probably performed with a certain fraction of V_B_^–^ in the metastable state, specifically with a reduced concentration of V_B_^–^. Reducing the probe current to below 1 pA and increasing the exposure time would resolve this issue, although it is expected to result in a lower signal-to-noise ratio of the spectrum, making measurements more challenging. Alternatively, when the τ of V_B_^0^ is comparable to that of V_B_^–^, the V_B_^0^/V_B_^–^ ratio can be considered quantitative despite the underestimation of the respective absolute densities. In any case, the optimization of EELS conditions and a more precise quantitative evaluation of both relative and absolute densities remain future challenges.

In summary, in this study, we investigated the intricate characteristics of optically active V_B_^–^ and optically inactive V_B_^0^ defects in nitrogen-ion irradiated h-BN by STEM–EELS with monochromated 30-keV electrons and first-principles simulations. EELS played a pivotal role in identifying the subgap states resulting from the irradiation, distinguishing distinct spectral peaks at 2.5 and 1.9 eV corresponding to V_B_^–^ and V_B_^0^ defects, respectively. The concentrations of V_B_^–^ and V_B_^0^ defects were estimated as approximately 300 and 1700 ppm on average, respectively. We also accomplished the concentration mapping of these defects at the nanometer scale, which revealed their near-uniform distribution without significant segregations. As a future challenge, it is necessary to optimize EELS conditions by considering the lifetime τ to avoid the underestimation of defect concentration. Such optimization is imperative for precise quantitative assessments, as it provides indispensable insights that are vital for the future application of h-BN in quantum technology sectors.

## Supplementary Material



## Notes

Details of experimental method, first-principles simulations, other possible defects, loss function calculation, and additional figures (PDF)
