# Supplementary material for: Observation of Boron Vacancy Concentration in Hexagonal Boron Nitride at Nanometer Scale
Source: Nano Lett. 2025 Aug 20;25(35):13191–7. doi: 10.1021/acs.nanolett.5c02988 (PMC12412148; doi:10.1021/acs.nanolett.5c02988)
Supplement: Supplementary file 1 [file nl5c02988_si_001.pdf]

Supporting information

Observation of Boron Vacancy Concentration in Hexagonal Boron Nitride at Nanometer Scale

Jun Kikkawa,[*,†] Chikara Shinei,*[†] Jun Chen,*[†] Yuta Masuyama,*[‡] Yuichi Yamazaki,*[‡] Teruyasu Mizoguchi,*[§] Koji Kimoto,*[†] Takashi Taniguchi,*[†] and Tokuyuki Teraji*[†]

[†] National Institute for Materials Science, 1-1 Namiki, Tsukuba 305-0044, Japan
[‡] National Institutes for Quantum Science and Technology, 1233 Watanukimachi, Takasaki 370-1292, Japan
[§] Institute of Industrial Science, The University of Tokyo, 4-6-1 Komaba, Meguro, Tokyo 153-8505, Japan
*E-mail: kikkawa.jun@nims.go.jp

Contents

I. Methods
    A. Experiments
    B. First-principles simulations
II. Other possible defects
III. Calculation of loss function
IV. Supplemental figures: S1–S10

List of abbreviations

h-BN: hexagonal boron nitride
HPHT: high pressure and high temperature
PL: photoluminescence
ODMR: optically detected magnetic resonance
CL: cathodoluminescence
EELS: electron energy loss spectroscopy
ZLP: zero-loss peak
BG: background
DOS: density of states
CBM: conduction band minimum
VBM: valence band maximum
ZPL: zero-phonon line

I. Methods

A. Experiments

h-BN single crystals were obtained using a temperature gradient method at HPHT of 4.0 GPa and 1600 °C, respectively, with a Ba–BN solvent.[1] Flakes with a thickness of 30–200 nm were prepared from an h-BN crystal by a tape-peeling method using a water-soluble tape. The h-BN flakes were detached from the tape by ultrasonication in pure water and washed in ethanol. The flakes were then dispersed on a holey carbon-film-supported copper grid (Quantifoil Micro Tools GmbH) with the *c*-axis orientation. The entire grid was irradiated with a 40-keV nitrogen ion (N_2^+) beam at a total dose of 1×10^{15} cm^{-2} (=10 nm^{-2}) at room temperature.[2] PL was measured at room temperature using a microspectroscopy system (Nanofinder FLEX; Tokyo Instruments, Inc.) with a photon energy of 2.33 eV (=532 nm). ODMR was measured at room temperature using a home-made confocal microscope with 532 nm excitation laser.[2] For CL spectroscopy, we used a CL system (MP-32; Horiba Ltd.) attached to a scanning electron microscope (SU6600; Hitachi High-Tech Co.). CL spectra were acquired from free-standing h-BN regions (i.e., hole regions of the carbon film) using 5 keV electrons at room temperature. For EELS, we used a monochromated transmission electron microscope (Themis Z; Thermo Fisher Scientific Inc.) equipped with a spectrometer (Quantum 970, Gatan Inc.). A CCD camera (994 US1000XP U+, Gatan Inc.) optimized for 30 keV electrons was used,[3] reducing the readout noise.[4, 5] The energy of the electron probe was set to 30 keV to suppress Cherenkov radiation.[6, 7] The probe diameter and current were 0.6−0.7 nm and ~120 pA, respectively. The EELS spectra in Figures S1a and 1d were collected in the 105−120-nm-square areas of free-standing regions in scan steps of 1.1−1.8 nm with dwell times of 0.02 s (Figure S1a) and 0.1 s (Figure 1d), and they were summed to obtain a single spectrum after the subtraction of dark noise and the alignment of the zero-loss peak (i.e., elastic scattering peak). For the EELS spectrum in Figures 1e, S3, and S4, the collected area, scan step, and dwell time were 143 nm square, 3.6 nm, and 0.6 s, respectively. The convergence semiangle α was set to 13.9 mrad (Figures S1a and 1c) and 12.5 mrad (Figure 1d). The collection semiangle β was set to 13.9 mrad (= 12.5 nm^{-1}; Figures S1a and 1d) and 15.3 mrad (= 13.8 nm^{-1}; Figures 1e, S3 and S4), which were larger than the magnitude of $\overrightarrow{\mathrm{KM}}$ (8.36 nm^{-1}), enabling the acceptance of the excited electrons from the VBM at the K-point to the CBM at the M-point. The FWHM values of ZLP were 100 meV (Figure S1), 65 meV (Figure 1d), and 40 meV (Figures 1e, S3 and S4). For the B- and N-*K* edges, α and β were set to 13.9 and 34.8 mrad, respectively (Figures S1b and S1c). The thickness of the h-BN flakes was estimated to be 30–200 nm (i.e., 129 and 159

nm for pristine and irradiated h-BN, respectively, in Figures 1d, 1e, S1, S3, and S4), using the standard log-ratio method.[8] All EELS spectra were acquired at room temperature.

B. Frist-principles simulations

First-principles simulations were performed on the basis of density functional theory using the projector augmented wave method, as implemented in the VASP code.[9] The geometries of the V_B^0 and V_B^- defect models were optimized. The supercell consisted of 72 atoms, constructed by expanding the primitive cell of h-BN by a factor of 3×3×2. An energy cutoff of 500 eV and a Monkhorst–Pack *k*-point mesh of 3×3×2 were employed. Structural relaxations were carried out until the residual forces on all atoms were reduced below 0.08 eV/Å. Given that h-BN is a van der Waals layered material, dispersion interactions were considered using the rev-vdW-DF2 functional,[10] which accurately reproduces lattice parameters of layered systems. Spin polarization was included in all calculations. After obtaining optimized structures for the V_B^0 and V_B^- defect models, electronic structure calculations were performed. Although accurate descriptions of in-gap states often require advanced methods such as hybrid functionals or GW approximations, these are computationally demanding for large supercells. Therefore, we employed the modified Becke–Johnson (mBJ) exchange potential, a meta-GGA functional known for its efficiency and capability to yield band gaps comparable to those obtained from more rigorous methods. The original mBJ potential, which was proposed by Becke and Johnson,[11] was later modified by Tran and Blaha to better reproduce band gap properties of semiconductors and insulators.[12] Using the optimized structures of the perfect crystal model, and the V_B^0 and V_B^- defect models, we calculated their electronic structures with the mBJ potential. The optical properties, specifically the frequency-dependent dielectric function, were evaluated within the independent-particle random phase approximation based on the mBJ functional. In these simulations, both excitonic effects and local-field corrections were neglected.

II. Other possible defects

As reviewed by Wu et al.,[13] C_N, C_B, and C_BC_N (carbon dimer) defects are intrinsically present in greater quantities in h-BN. The C_BC_N defect is a strong candidate for the luminescence observed at 4.1 eV,[14, 15] which has been detected in both commercial and HPHT h-BN.[16, 17] For HPHT h-BN, the 4.1 eV luminescence appears predominantly in C-rich regions.[18] Consistently, in this study, we observed CL at 4.08 eV, independent of irradiation (Figure S2). The luminescence at 3.22 eV, previously reported in both commercial and HPHT h-BN, is associated with C_N and C_B defects.[13, 19] In HPHT h-BN, the 3.22 eV CL is notably weak in non-doped samples. [20] With increasing levels of intentional carbon doping, the 4.1 eV CL emerges at lower doping levels, while the 3.22 eV CL appears only at higher doping concentrations.[20] In the non-doped HPHT h-BN used in this study, only a weak 4.08 eV CL was observed (Figure S2), indicating the presence of C_BC_N defects, whereas the concentrations of C_N and C_B defects are negligible. Regarding other types of defects, PL at 1.97–1.99 eV, attributed to a ZPL with phonon sidebands between 1.67 and 1.83 eV, has been reported in commercial h-BN.[21, 22] These defects are believed to be either N_BV_N or C_BV_N.[21, 23, 24] PL at 2.08 eV, also attributed to the ZPL, with phonon sidebands at 1.92 eV and 1.77 eV, has additionally been reported and ascribed to the carbon trimer C_NC_2.[25] It has also been shown that a ZPL around 2 eV can appear in two oppositely charged monomers, C_B^+–C_N^-, separated by some distance.[25] In the HPHT h-BN flakes used in this study, if defects responsible for the 2 eV luminescence were present in significant amounts, phonon sideband signals should be observable in the PL spectrum around 1.7–1.9 eV. However, no such features were detected (Figure 1b). Therefore, the concentrations of such complex defects (e.g., C_BV_N, C_NC_2, $C_B^+ - C_N^-$, etc.) can be considered negligible. Notably, even in intentionally C-doped HPHT h-BN, 2 eV luminescence does not appear.[20] In HPHT h-BN, with increasing C concentration, C_BC_N defects, which are responsible for the 4.1 eV luminescence, are preferentially formed, followed by C_N and C_B defects, which give rise to the 3.22 eV luminescence. Additionally, the formation energy of interstitial carbon C_i is theoretically predicted to be higher than those of C_N or C_B,[26] rendering its presence unlikely. Thus, it is reasonable to conclude that in the pristine h-BN flakes used in this study, only a small quantity of C_BC_N is present prior to irradiation. Furthermore, the fact that neither the 4.1 eV nor the 3.22 eV CL increases after the irradiation (Figures S2) indicates that the state of carbon-related defects is not significantly affected by the irradiation process. The EELS signal originating from C_BC_N defects is expected to appear above 4.1 eV, and thus is outside the energy range of interest in this study (i.e., 1.5–3.5 eV). In our non-doped HPHT h-BN, the oxygen concentration

(<1 ppm) is an order of magnitude lower than the carbon concentration (<10 ppm).[27] Although O_N defects are expected to be present due to their low formation energy,[26] luminescence at 3.4 eV, which has been attributed to O_N defects,[28] was not observed in the CL spectra (Figure S2). Given that the defect level associated with O_N defects is thought to be close to the CBM, no EELS signal from O_N is expected in the energy range relevant to this study (i.e., 1.5–3.5 eV).

It is therefore reasonable to consider that the defect formation resulting from atomic displacements induced by ion irradiation is the predominant process, rather than the intrinsic defects associated with carbon or oxygen. Although the detailed mechanisms of defect formation under ion irradiation (e.g., effects of the species, energy, flux, and total dose of the charged particle beam) have not been fully elucidated, it is widely accepted that vacancies (i.e., V_B and V_N) generated in the primary process are the main defects. Direct observations have shown that electron and He^+ ion irradiation preferentially generate V_B defects rather than V_N defects.[29-31] Because the formation of anti-site defects (i.e., B_N and N_B) requires secondary processes, their formation probability is substantially lower. Therefore, in addition to the formation of V_B and V_N defects, the primary irradiation products are considered to include B_i and N_i interstitial atoms generated during these processes. Given the relatively low migration barriers for all stable charge states of B_i and N_i, these atoms can migrate to vacancy sites, resulting in lattice restoration or, less commonly, the formation of anti-sites, and they can also migrate to step edges even at room temperature.[26] Thus, isolated B_i and N_i atoms are highly unlikely to be present as stable defects under the conditions of this study.

Previous studies have examined the effects of N_2^+ and Ar^+ ion irradiation on h-BN, focusing on the B- and N-*K* edges, and suggested that the formation of N_i atoms is associated with the creation of V_N defects or that aggregation of N_i atoms leads to N_2 molecules.[32-34] However, in this study, we observed no marked changes at the N *K* edge after the irradiation; only a slightly asymmetric broadening of the peak at 191.8 eV was detected at the B *K* edge (Figures S1b and S1c). These findings demonstrate that irradiation damage in our specimens remains at an early stage, in contrast to previous reports.[32-34] The observed broadening on the low energy-loss side of the B *K* edge at 191.8 eV (Figure S1b) does not indicate the formation of N_i atoms or N_2 molecules, but is instead attributed to the formation of V_N^0 defects,[19] which introduce defect levels just below the CBM,[26, 35] consistent with our observations.

In summary, in addition to the V_B^- and V_B^0 defects, it is recognized that V_N^0 and C_BC_N defects may also coexist in the h-BN flakes after the irradiation used in this study. However, as the energy levels associated with these defects lie outside the energy range

of interest (i.e., 1.5–3.5 eV), it is appropriate to focus exclusively on the V_B^- and V_B^0 defects when evaluating their concentrations.

III. Calculation of loss function

In uniaxial materials, the dielectric tensor $\boldsymbol{\varepsilon}_{ij}$ is written as

$$\boldsymbol{\varepsilon}_{ij}=\begin{pmatrix}\boldsymbol{\varepsilon}_{xx} & 0 & 0\\ 0 & \boldsymbol{\varepsilon}_{yy} & 0\\ 0 & 0 & \boldsymbol{\varepsilon}_{zz}\end{pmatrix},$$

where $\boldsymbol{\varepsilon}_{xx}=\boldsymbol{\varepsilon}_{yy}$, $\boldsymbol{\varepsilon}_{xx}=\varepsilon_{1,xx}+i\varepsilon_{2,xx}$, and $\boldsymbol{\varepsilon}_{zz}=\varepsilon_{1,zz}+i\varepsilon_{2,zz}$. The loss function L, when the electron incident direction is parallel to the c-axis and convergence angle α=0, is calculated as[36]

$$L=-\mathrm{Im}\left[\frac{1}{2\boldsymbol{\varepsilon}_{zz}}\ln\left(1+\frac{\boldsymbol{\varepsilon}_{zz}\beta^2}{\boldsymbol{\varepsilon}_{xx}\theta_E^{\,2}}\right)\right].$$

β and θ_{E} are the collection and characteristic angles, respectively. $\theta_E=\Delta E/2E_0$ in nonrelativistic form, where E_0 and ΔE represent the incident energy and energy loss of the primary electron, respectively: $\theta_{\mathrm{E}}=4.2\times10^{-2}$ mrad for E_0=30 keV and ΔE=2.5 eV. By defining $u=\beta/\theta_E$, we can expand the equation for L as follows.

$$L=\frac{1}{2}\frac{\varepsilon_{2,zz}}{\varepsilon_{1,zz}{}^2+\varepsilon_{2,zz}{}^2}\ln\left[1+\frac{(\varepsilon_{1,zz}+\mathrm{i}\varepsilon_{2,zz})}{\varepsilon_{1,xx}+\mathrm{i}\varepsilon_{2,xx}}u^2\right]$$

$$=\frac{1}{4}\frac{\varepsilon_{2,zz}}{\varepsilon_{1,zz}{}^2+\varepsilon_{2,zz}{}^2}\ln\left[\left(1+\frac{\varepsilon_{1,xx}\varepsilon_{1,zz}+\varepsilon_{2,xx}\varepsilon_{2,zz}}{\varepsilon_{1,xx}{}^2+\varepsilon_{2,xx}{}^2}u^2\right)^2+\left(\frac{\varepsilon_{1,xx}\varepsilon_{2,zz}-\varepsilon_{2,xx}\varepsilon_{2,zz}}{\varepsilon_{1,xx}{}^2+\varepsilon_{2,xx}{}^2}u^2\right)^2\right]$$

In the case where the electron beam has the convergence angle α ($\neq$0), β can be replaced with the effective collection angle $\beta^*=\sqrt{\alpha^2+\beta^2}$.[8, 37]

IV. Supplemental figures

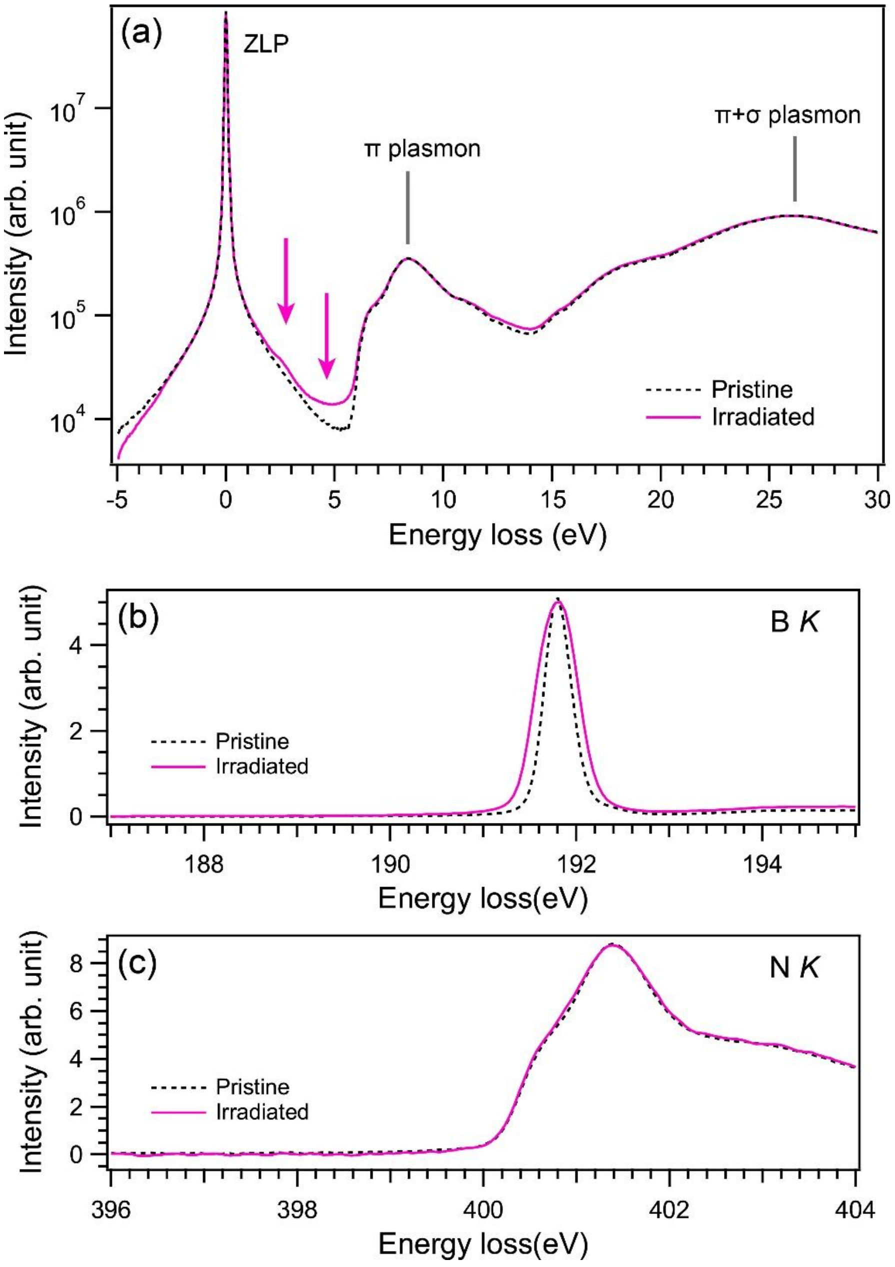

Figure S1. EELS spectra of pristine (dashed line) and irradiated (solid line) h-BN flakes. (a) ZLP, bandgap, π plasmon, and π+σ plasmon. Arrows in (a) indicate extra intensities observed only after the irradiation. (b) B *K* edge. The peak width at 191.8 eV increased after the irradiation. (c) N *K* edge.

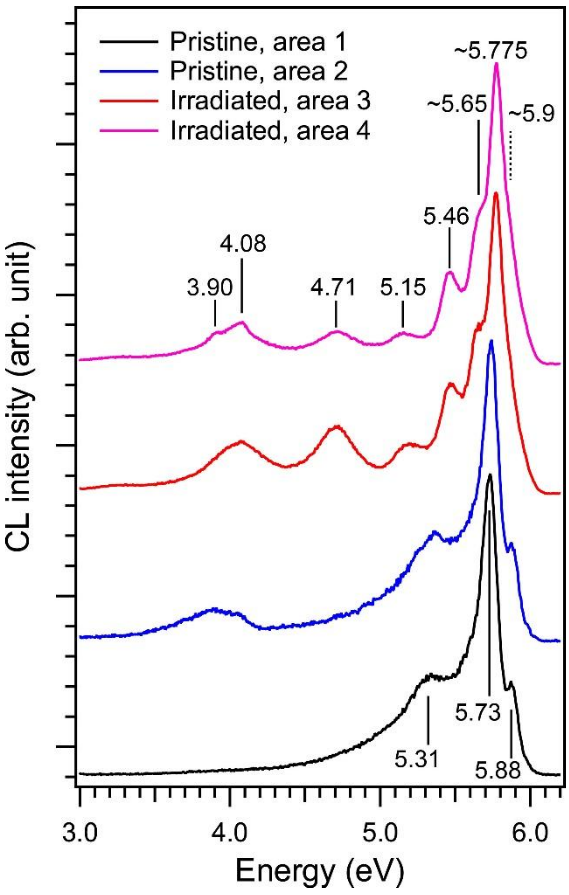

Figure S2. CL spectra of pristine and irradiated h-BN flakes. For the pristine flake, intensities at 5.88 and 5.73 eV are attributed to intrinsic excitons.[38] For the irradiated flake, intensities at ~5.9, 5.78, 5.65, and 5.46 eV are likely excitons. The intensities at 4.08 and 3.90 eV are attributed to carbon-based defects,[14-16] which are probably introduced inhomogeneously during the growth of h-BN single crystals.

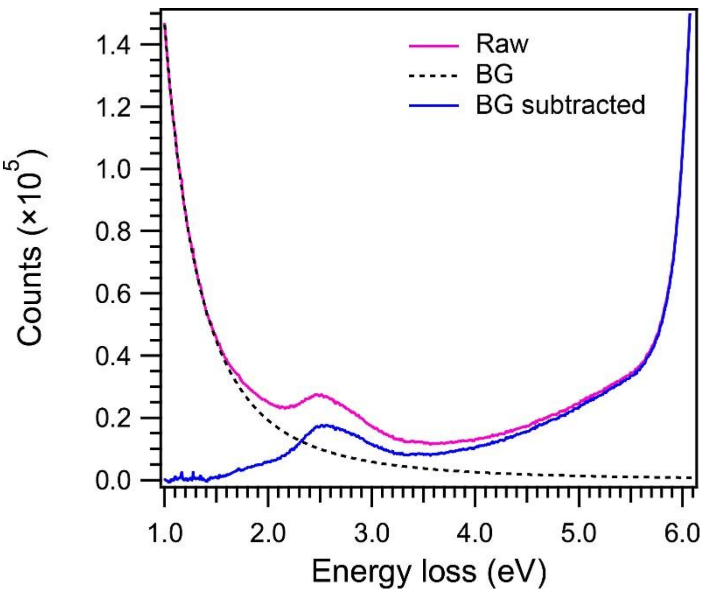

Figure S3. Subtraction of the BG intensity (i.e., ZLP tail) using a power-low fit (dashed line) from the raw EELS spectrum.

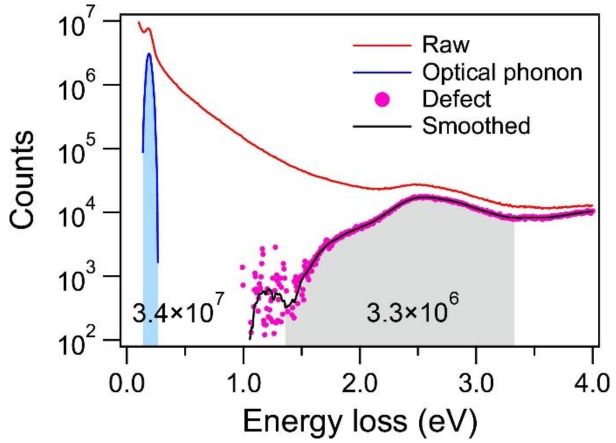

Figure S4. Comparison of the integrated intensities (filled regions) between the optical phonon peak at 0.19 eV (3.4×10^7 counts) and the defect-related asymmetric peak at 2.5 eV (3.3×10^6 counts) after subtraction of the BG intensity (i.e., ZLP tail) from the raw EELS spectrum. The defect-related peak (solid circle) is identical to the profile (BG subtracted) in Figure S2, and its smoothed profile is also shown. The integrated ranges for the optical phonon and defect-related peak are 0.13–0.28 eV and 1.34–3.34 eV, respectively.

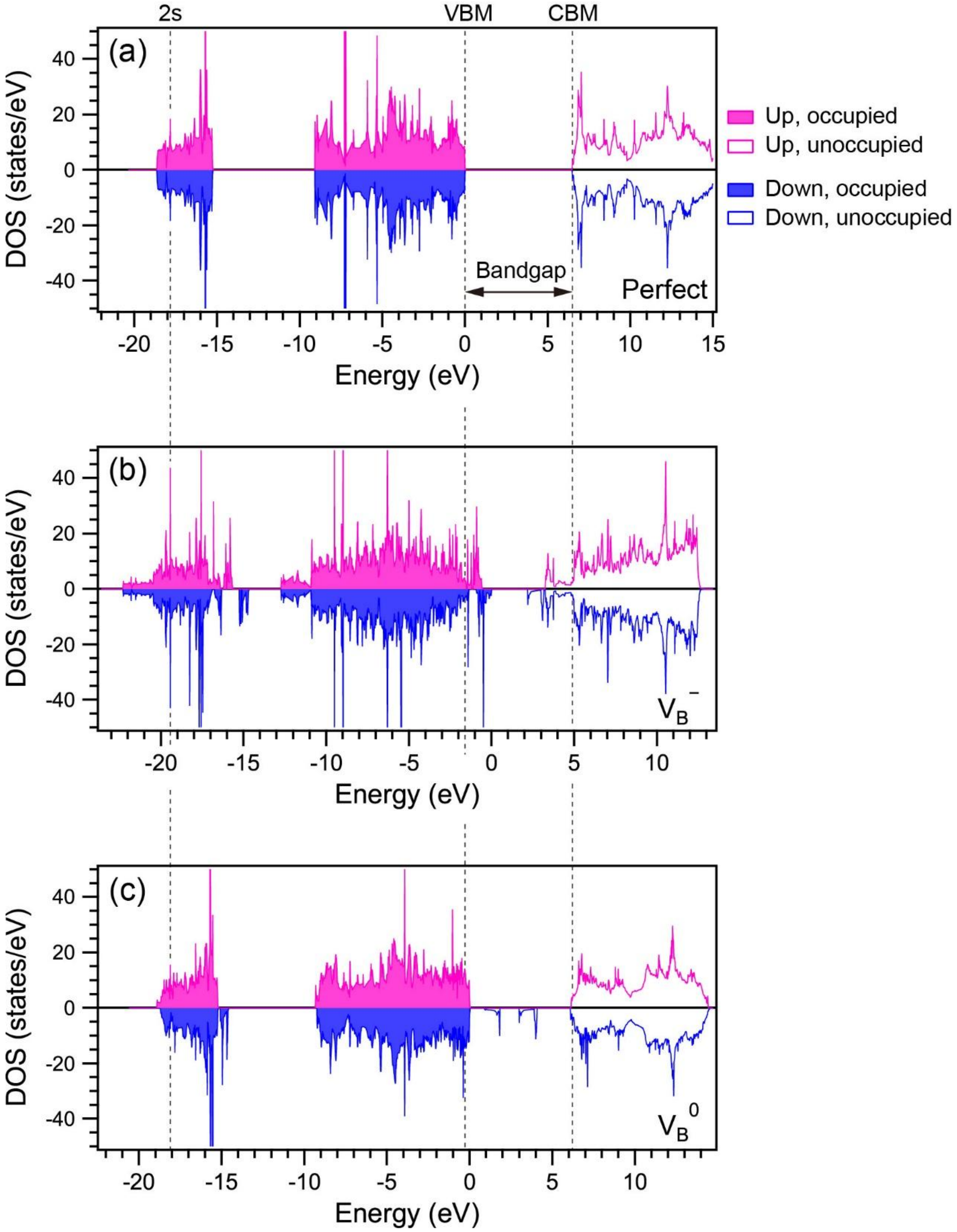

Figure S5. DOS values of h-BN crystals without defects, with a V_B^- defect, and with a V_B^0 defect. The filled and blank areas denote occupied and unoccupied states, respectively. The upper and lower sides represent up- and down-spin states, respectively. The three DOS diagrams are aligned with the 2s levels.

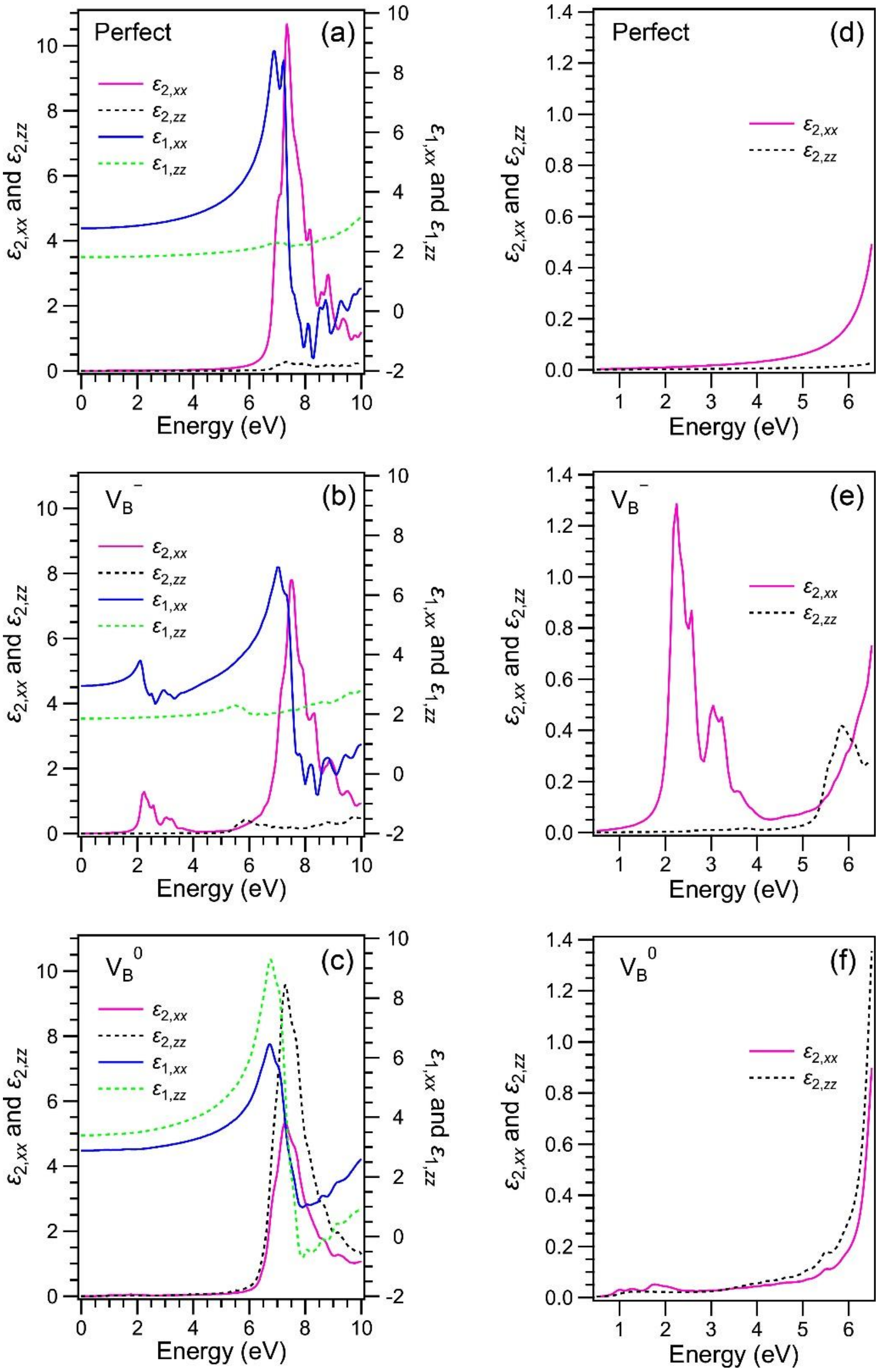

Figure S6. Calculated dielectric functions for the imaginary parts $\varepsilon_{2,xx}$ and $\varepsilon_{2,zz}$ and the real parts $\varepsilon_{1,xx}$ and $\varepsilon_{1,zz}$, for the perfect crystal in (a), V_B^- in (b), and V_B^0 in (c). The imaginary parts $\varepsilon_{2,xx}$ and $\varepsilon_{2,zz}$ between 0.5 and 6.5 eV in (a)–(c) are enlarged in (d)–(f), respectively.

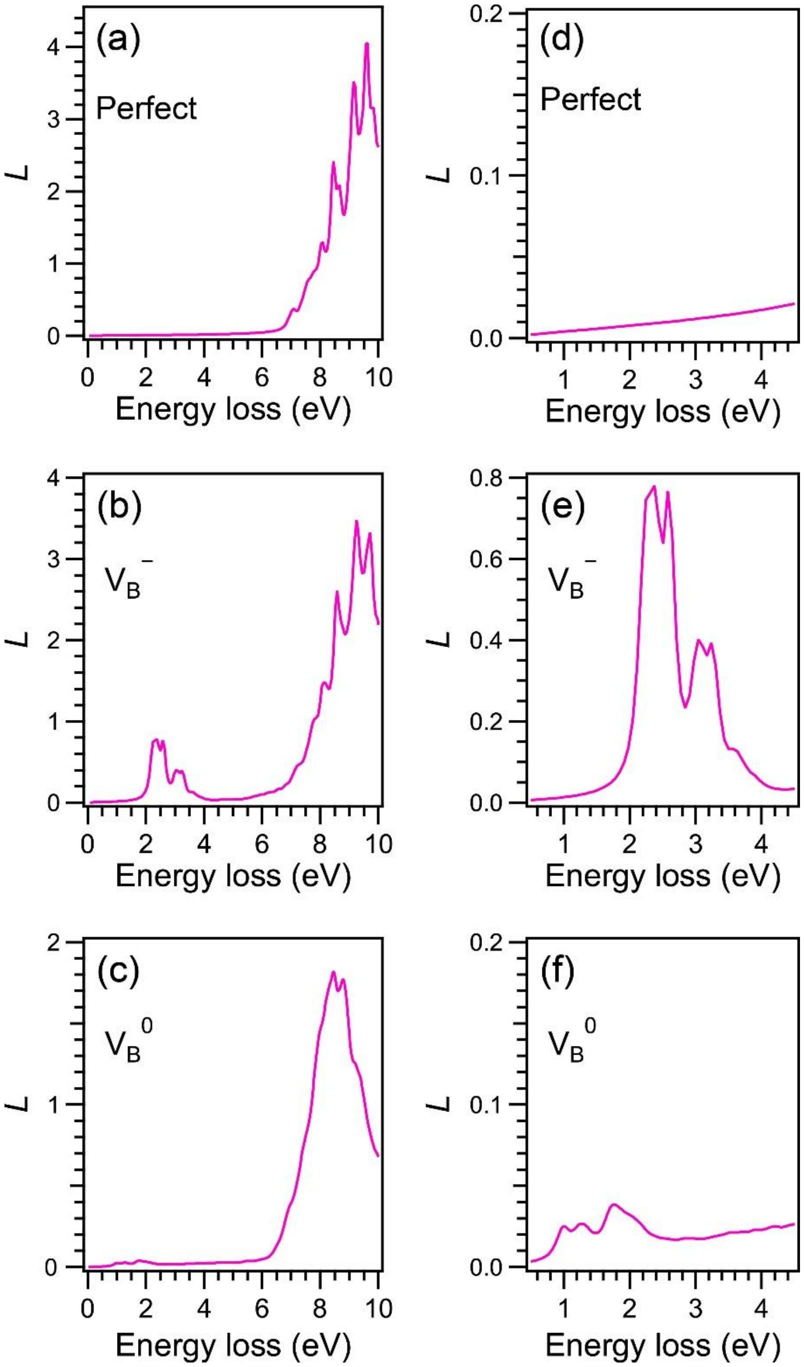

Figure S7. Calculated loss function L for perfect in (a), V_B^- in (b), and V_B^0 in (c). L between 0.5 and 4.5 eV in (a)–(c) are enlarged in (d)–(f), respectively.

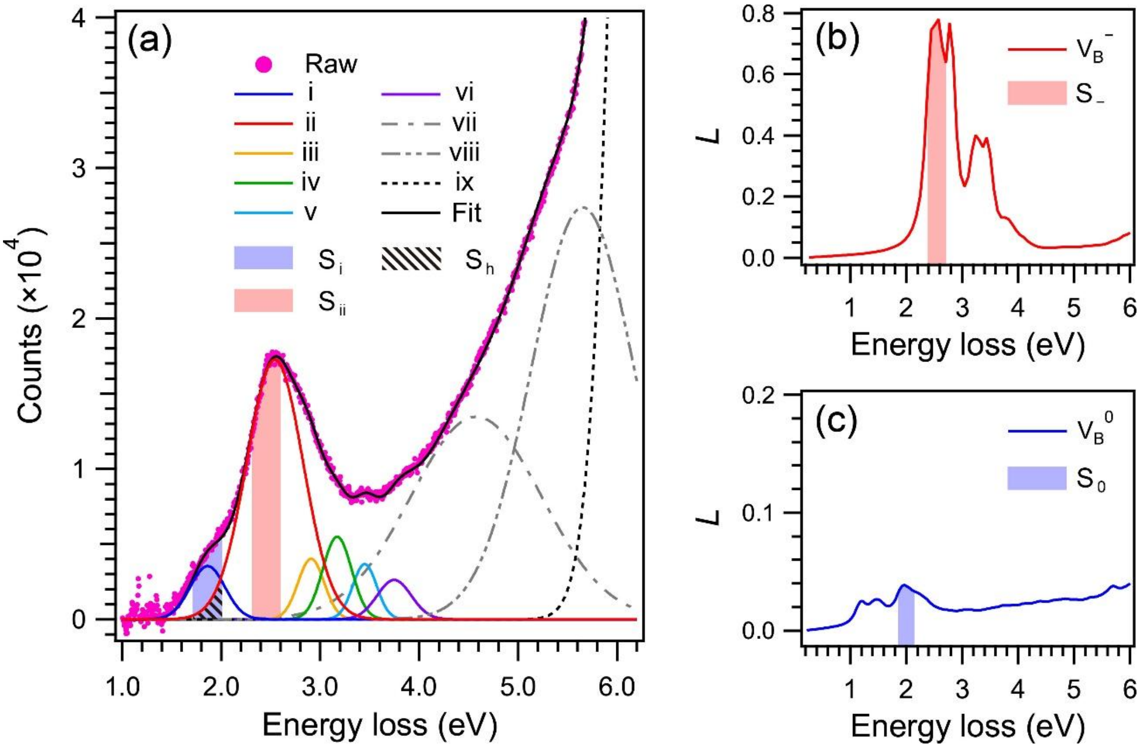

Figure S8. Evaluation of the average $V_B{}^0/V_B{}^-$ concentration ratio. (a) Multiple Gaussian fits for the EELS spectrum after subtraction of the ZLP tail in Figure S3. [(b) and (c)] Plots of the loss functions L ($V_B{}^-$) in (b) and L ($V_B{}^0$) in (c) with an energy shift of +0.2 eV for comparison with the experimental EELS spectrum. Gaussian profiles i–vi are related to the peaks A' –J' in L ($V_B{}^-$) and L ($V_B{}^0$) as follows: i is related to I' and J', ii to A' and B', iii to C', iv to D', v to E', and vi to F'. The sum of all Gaussian profiles i–x is denoted by the profile fit in (a). The filled areas S_i and S_{ii} in (a) denote the integrated intensity in the EELS spectrum with energy ranges of 1.7–2.0 eV and 2.3–2.6 eV, respectively, whereas the hatched area S_h denotes the overlapping area of Gaussian profiles i and ii. The filled areas S_- in (b) and S_0 in (c) denote the integrated intensity in L ($V_B{}^-$) and L ($V_B{}^0$) with an energy width of 0.3 eV. Setting the equation $(S_i - S_h)/S_{ii} = \eta S_0/S_-$, the coefficient η is evaluated as 5.3. Then, the coefficient values of $1/(1+\eta)$=0.16 for L ($V_B{}^-$) and $\eta/(1+\eta)$=0.84 for L ($V_B{}^0$) in Figure 3 were obtained.

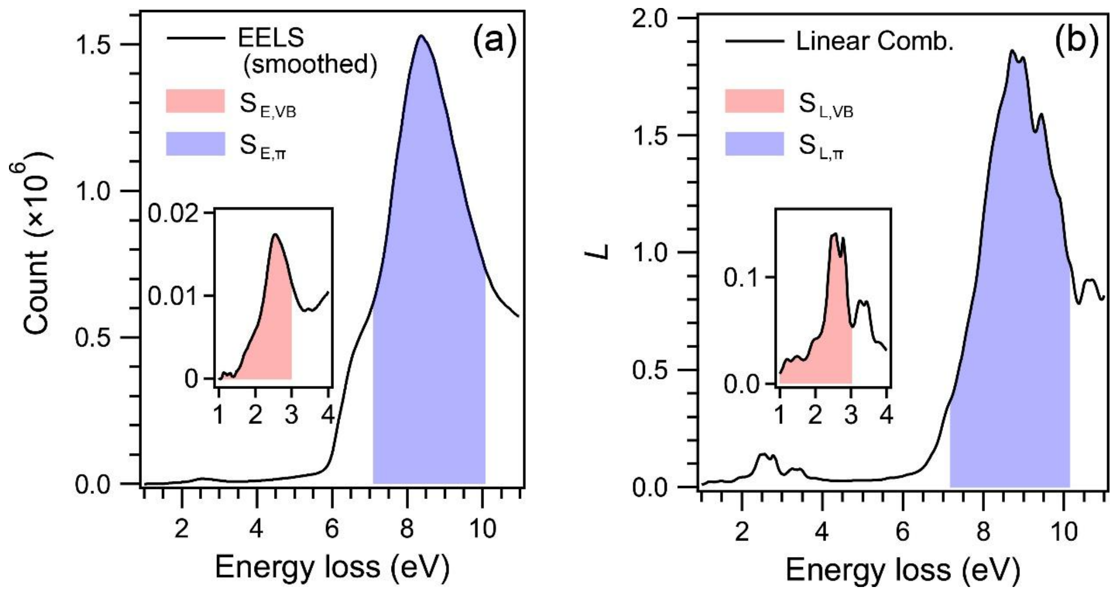

Figure S9. Evaluation of the average joint concentration of V_B^- and V_B^0 defects. (a) Smoothed profile of the EELS spectrum with a wider energy range in Figure 3. (b) Linear combination profile, 0.16 L (V_B^-) + 0.84 L (V_B^0), in Figure 3. The inset profiles in (a) and (b) enlarge the energy region of 1−4 eV. The filled areas $S_{E,VB}$ in (a) and $S_{L,VB}$ in (b) denote the integrated intensity for V_B^- and V_B^0 defects with a 2.0 eV energy width, whereas $S_{E,\pi}$ in (a) and $S_{L,\pi}$ in (b) denote the integrated intensity for the π plasmon with a 3.0 eV energy width. By setting the equation $n/m = (S_{E,VB}/S_{E,\pi})/(S_{L,VB}/S_{L,\pi})$ with a defect concentration in the supercell used in first-principles simulations, m=13889 ppm, we evaluated the joint concentration of V_B^- and V_B^0, n, as 2000 ppm Then, the average concentrations 0.16n=320 ≈300 ppm for V_B^- and 0.84n= 1680 ≈1700 ppm for V_B^0 were obtained.

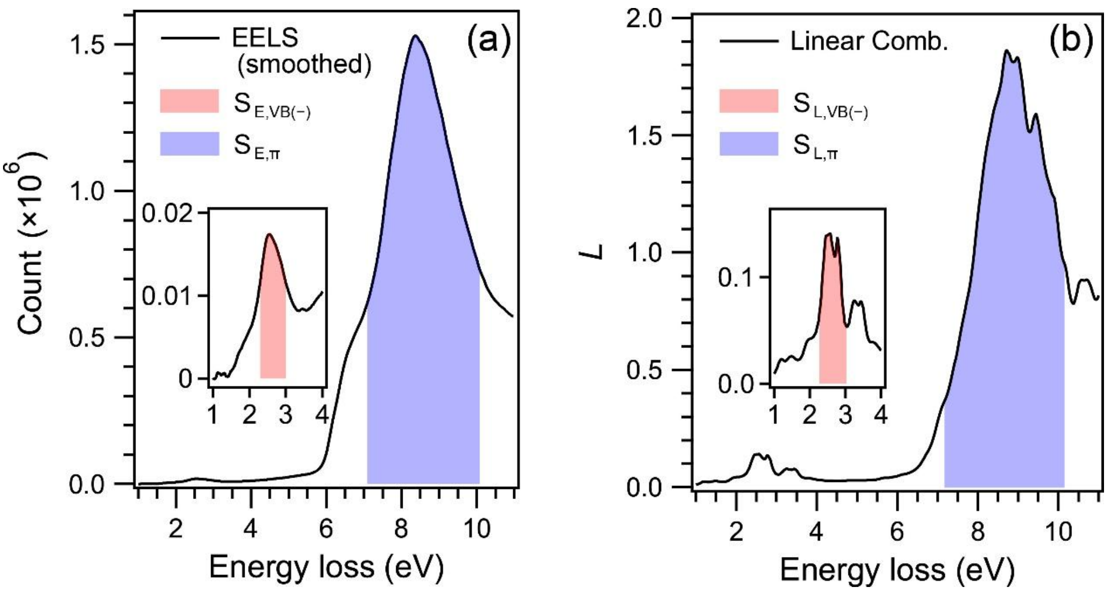

Figure S10. Direct evaluation of the concentration of V_B^- defects. (a) Smoothed profile of the EELS spectrum with a wider energy range in Figure 3. (b) Linear combination profile, $0.16L\,(V_B^-) + 0.84L\,(V_B^0)$, in Figure 3. The inset profiles in (a) and (b) enlarge the energy region of 1−4 eV. The filled areas $S_{E,VB(-)}$ in (a) and $S_{L,VB(-)}$ in (b) denote the integrated intensity for V_B^- defects with a 0.7 eV energy width, whereas $S_{E,\pi}$ in (a) and $S_{L,\pi}$ in (b) denote the integrated intensity for the π plasmon with a 3.0 eV energy width, as in Figure S9. Setting the equation $0.16n/0.16m = (S_{E,VB(-)}/S_{E,\pi})/(S_{L,VB(-)}/S_{L,\pi})$, we evaluated the concentration of V_B^- as $0.16n$=337≈300 ppm, which corresponds to that estimated using $S_{E,\pi}$ and $S_{L,\pi}$ in Figure S9. The V_B^- map in Figure 5a was generated using the equation $0.16n/0.16m = (S_{E,VB(-)}/S_{E,\pi})/(S_{L,VB(-)}/S_{L,\pi})$ at each pixel.

References

(1) Taniguchi, T.; Watanabe, K. Synthesis of High-Purity Boron Nitride Single Crystals under High Pressure by Using Ba–BN Solvent. *J. Cryst. Growth* **2007**, *303* (2), 525–529.

(2) Suzuki, T.; Yamazaki, Y.; Taniguchi, T.; Watanabe, K.; Nishiya, Y.; Matsushita, Y.; Harii, K.; Masuyama, Y.; Hijikata, Y.; Ohshima, T. Spin Property Improvement of Boron Vacancy Defect in Hexagonal Boron Nitride by Thermal Treatment. *Appl. Phys. Express* **2023**, *16* (3), 032006.

(3) Kikkawa, J.; Taniguchi, T.; Kimoto, K. Nanometric Phonon Spectroscopy for Diamond and Cubic Boron Nitride. *Phys. Rev. B* **2021**, *104* (20), L201402.

(4) Haruta, M.; Fujiyoshi, Y.; Nemoto, T.; Ishizuka, A.; Ishizuka, K.; Kurata, H. Extremely Low Count Detection for EELS Spectrum Imaging by Reducing CCD Read-out Noise. *Ultramicroscopy* **2019**, *207*, 112827.

(5) Haruta, M.; Kikkawa, J.; Kimoto, K.; Kurata, H. Comparison of Detection Limits of Direct-Counting CMOS and CCD Cameras in EELS Experiments. *Ultramicroscopy* **2022**, *240*, 113577.

(6) Chen, C. H.; Silcox, J. Calculations of the Electron-Energy-Loss Probability in Thin Uniaxial Crystals at Oblique Incidence. *Phys. Rev. B* **1979**, *20* (9), 3605–3614.

(7) Laturia, A.; Van de Put, M. L.; Vandenberghe, W. G. Dielectric Properties of Hexagonal Boron Nitride and Transition Metal Dichalcogenides: From Monolayer to Bulk. *npj 2D Mater. Appl.* **2018**, *2* (1), 6.

(8) Egerton, R. F. *Electron Energy-Loss Spectroscopy in the Electron Microscope*; Springer Science+Business Media, LLC, 2011.

(9) Kresse, G.; Joubert, D. From Ultrasoft Pseudopotentials to the Projector Augmented-Wave Method. *Phys. Rev. B* **1999**, *59* (3), 1758–1775.

(10) Hamada, I. van der Waals Density Functional Made Accurate. *Phys. Rev. B* **2014**, *89* (12), 121103.

(11) Becke, A. D.; Johnson, E. R. A Simple Effective Potential for Exchange. *J. Chem. Phys.* **2006**, *124* (22).

(12) Tran, F.; Blaha, P. Accurate Band Gaps of Semiconductors and Insulators with a Semilocal Exchange-Correlation Potential. *Phys. Rev. Lett.* **2009**, *102* (22), 226401.

(13) Wu, A.; Badrtdinov, D. I.; Lee, W.; Rösner, M.; Dreyer, C. E.; Koperski, M. Ab Initio Methods Applied to Carbon-Containing Defects in Hexagonal Boron Nitride. *Mater. Today Sustain.* **2024**, *28*, 100988.

(14) Plo, J.; Pershin, A.; Li, S.; Poirier, T.; Janzen, E.; Schutte, H.; Tian, M.; Wynn, M.; Bernard, S.; Rousseau, A.; et al. Isotope Substitution and Polytype Control for Point

Defects Identification: The Case of the Ultraviolet Color Center in Hexagonal Boron Nitride. *Phys. Rev. X* **2025**, *15* (2), 021045.
(15) Mackoit-Sinkevičienė, M.; Maciaszek, M.; Van de Walle, C. G.; Alkauskas, A. Carbon Dimer Defect as a Source of the 4.1 eV Luminescence in Hexagonal Boron Nitride. *Appl. Phys. Lett.* **2019**, *115* (21).
(16) Silly, M. G.; Jaffrennou, P.; Barjon, J.; Lauret, J. S.; Ducastelle, F.; Loiseau, A.; Obraztsova, E.; Attal-Tretout, B.; Rosencher, E. Luminescence Properties of Hexagonal Boron Nitride: Cathodoluminescence and Photoluminescence Spectroscopy Measurements. *Phys. Rev. B* **2007**, *75* (8), 085205.
(17) Gale, A.; Li, C.; Chen, Y.; Watanabe, K.; Taniguchi, T.; Aharonovich, I.; Toth, M. Site-Specific Fabrication of Blue Quantum Emitters in Hexagonal Boron Nitride. *ACS Photonics* **2022**, *9* (6), 2170-2177.
(18) Onodera, M.; Watanabe, K.; Isayama, M.; Arai, M.; Masubuchi, S.; Moriya, R.; Taniguchi, T.; Machida, T. Carbon-Rich Domain in Hexagonal Boron Nitride: Carrier Mobility Degradation and Anomalous Bending of the Landau Fan Diagram in Adjacent Graphene. *Nano Lett.* **2019**, *19* (10), 7282–7286.
(19) McDougall, N. L.; Partridge, J. G.; Nicholls, R. J.; Russo, S. P.; McCulloch, D. G. Influence of Point Defects on the near Edge Structure of Hexagonal Boron Nitride. *Phys. Rev. B* **2017**, *96* (14), 144106.
(20) Ngamprapawat, S.; Kawase, J.; Nishimura, T.; Watanabe, K.; Taniguchi, T.; Nagashio, K. From h-BN to Graphene: Characterizations of Hybrid Carbon-Doped h-BN for Applications in Electronic and Optoelectronic Devices. *Adv. Electron. Mater.* **2023**, *9* (8), 2300083.
(21) Tran, T. T.; Bray, K.; Ford, M. J.; Toth, M.; Aharonovich, I. Quantum Emission from Hexagonal Boron Nitride Monolayers. *Nat. Nanotech.* **2016**, *11* (1), 37–41.
(22) Martínez, L. J.; Pelini, T.; Waselowski, V.; Maze, J. R.; Gil, B.; Cassabois, G.; Jacques, V. Efficient Single Photon Emission from a High-Purity Hexagonal Boron Nitride Crystal. *Phys. Rev. B* **2016**, *94* (12), 121405.
(23) Tawfik, S. A.; Ali, S.; Fronzi, M.; Kianinia, M.; Tran, T. T.; Stampfl, C.; Aharonovich, I.; Toth, M.; Ford, M. J. First-Principles Investigation of Quantum Emission from hBN Defects. *Nanoscale* **2017**, *9* (36), 13575–13582.
(24) Sajid, A.; Reimers, J. R.; Ford, M. J. Defect States in Hexagonal Boron Nitride: Assignments of Observed Properties and Prediction of Properties Relevant to Quantum Computation. *Phys. Rev. B* **2018**, *97* (6), 064101.
(25) Jara, C.; Rauch, T.; Botti, S.; Marques, M. A. L.; Norambuena, A.; Coto, R.; Castellanos-Águila, J. E.; Maze, J. R.; Munoz, F. First-Principles Identification of Single

Photon Emitters Based on Carbon Clusters in Hexagonal Boron Nitride. *J. Phys. Chem. A* **2021**, *125* (6), 1325–1335.
(26) Weston, L.; Wickramaratne, D.; Mackoit, M.; Alkauskas, A.; Van de Walle, C. G. Native Point Defects and Impurities in Hexagonal Boron Nitride. *Phys. Rev. B* **2018**, *97* (21), 214104.
(27) Ngamprapawat, S.; Nishimura, T.; Watanabe, K.; Taniguchi, T.; Nagashio, K. Current Injection into Single-Crystalline Carbon-Doped h-BN toward Electronic and Optoelectronic Applications. *ACS Appl. Mater. Interfaces* **2022**, *14* (22), 25731–25740.
(28) Grenadier, S. J.; Maity, A.; Li, J.; Lin, J. Y.; Jiang, H. X. Origin and Roles of Oxygen Impurities in Hexagonal Boron Nitride Epilayers. *Appl. Phys. Lett.* **2018**, *112* (16), 162103.
(29) Jin, C.; Lin, F.; Suenaga, K.; Iijima, S. Fabrication of a Freestanding Boron Nitride Single Layer and Its Defect Assignments. *Phys. Rev. Lett.* **2009**, *102* (19), 195505.
(30) Cretu, O.; Ishizuka, A.; Yanagisawa, K.; Ishizuka, K.; Kimoto, K. Atomic-Scale Electrical Field Mapping of Hexagonal Boron Nitride Defects. *ACS Nano* **2021**, *15* (3), 5316–5321.
(31) Liang, H.; Chen, Y.; Loh, L.; Cheng, N. L. Q.; Litvinov, D.; Yang, C.; Chen, Y.; Zhang, Z.; Watanabe, K.; Taniguchi, T.; et al. Site-Selective Creation of Blue Emitters in Hexagonal Boron Nitride. *ACS Nano* **2025**, *19* (15), 15130–15138.
(32) Jiménez, I.; Jankowski, A. F.; Terminello, L. J.; Sutherland, D. G. J.; Carlisle, J. A.; Doll, G. L.; Tong, W. M.; Shuh, D. K.; Himpsel, F. J. Core-Level Photoabsorption Study of Defects and Metastable Bonding Configurations in Boron Nitride. *Phys. Rev. B* **1997**, *55* (18), 12025–12037.
(33) Peter, R.; Bozanic, A.; Petravic, M.; Chen, Y.; Fan, L. J.; Yang, Y. W. Formation of Defects in Boron Nitride by Low Energy Ion Bombardment. *J. Appl. Phys.* **2009**, *106* (8), 083523.
(34) Bozanic, A.; Petravic, M.; Fan, L. J.; Yang, Y. W.; Chen, Y. Direct Observation of Defect Levels in Hexagonal BN by Soft X-Ray Absorption Spectroscopy. *Chem. Phys. Lett.* **2009**, *472* (4), 190–193.
(35) Strand, J.; Larcher, L.; Shluger, A. L. Properties of Intrinsic Point Defects and Dimers in Hexagonal Boron Nitride. *J. Phys.: Condens. Matter* **2020**, *32* (5), 055706.
(36) Browning, N. D.; Yuan, J.; Brown, L. M. Theoretical Determination of Angularly-Integrated Energy-Loss Functions for Anisotropic Materials. *Philos. Mag. A* **1993**, *67* (1), 261–271.
(37) Hage, F. S.; Ramasse, Q. M.; Kepaptsoglou, D. M.; Prytz, O.; Gunnaes, A. E.; Helgesen, G.; Brydson, R. Topologically Induced Confinement of Collective Modes in

Multilayer Graphene Nanocones Measured by Momentum-Resolved STEM–VEELS. *Phys. Rev. B* **2013**, *88* (15), 155408.
(38) Watanabe, K.; Taniguchi, T.; Kanda, H. Direct-Bandgap Properties and Evidence for Ultraviolet Lasing of Hexagonal Boron Nitride Single Crystal. *Nat. Mater.* **2004**, *3* (6), 404–409.
